# Guest editors' introduction to the Proceedings of the 9^th ^International Symposium on Biomedical Research and Applications

**DOI:** 10.1186/1471-2105-15-S13-S1

**Published:** 2014-11-13

**Authors:** Cynthia J Gibas, Zhipeng Cai, Oliver Eulenstein

**Affiliations:** 1 Department of Bioinformatics and Genomics, University of North Carolina at Charlotte, USA; 2 Department of Computer Science, Georgia State University, USA; 3 Department of Computer Science, Iowa State University, USA

## 

In these supplements to *BMC Bioinformatics *and *BMC Genomics*, we include a selection of research originally presented at the 9^th ^annual International Symposium on Bioinformatics Research Applications (ISBRA 2013). ISBRA 2013 was held at the University of North Carolina at Charlotte (Charlotte, NC, USA) on May 20-22, 2013.

For 9 years, the ISBRA symposium has been a forum for exchange of diverse ideas and research results broadly organized under the umbrella disciplines of bioinformatics and computational biology. The five keynote addresses at ISBRA 2013 included talks on RNA structure (Dr. Steve Harvey), computational behavioral ecology (Dr. Tanya Berger-Wolf), peptide identification from mass spectrometry (Dr. Bin Ma), gene regulation (Dr. Martha Bulyk), and biological network analysis (Dr. Luonan Chen). The research presented by conference participants was equally diverse. Reflecting current trends in the field, a substantial number of presentations were focused on genomics research, so we have chosen to present two linked supplements in *BMC Bioinformatics*, and one in *BMC Genomics*.

ISBRA 2013 was attended by 115 participants from the US, Canada, China, southeast Asia, and several European nations. Participants submitted 104 papers and 58 short abstracts. 25 papers were selected to appear in volume 7875 of Springer Verlag's Lecture Notes in the Bioinformatics [1] series, and four of these were revised and published as full length papers in a special supplement to the IEEE/ACM Transactions on Computational Biology and Bioinformatics [2]. 21 authors of short abstracts were invited to present short talks at the conference, and subsequently to expand on their work and submit full-length papers for these BMC Supplements. These submissions were subject to a rigorous peer review process and nine manuscripts have been selected for publication.

We thank the Program Committee members and Supplement external reviewers for volunteering their time to review and discuss symposium papers.

We thank the Steering Committee and Chairs of the ISBRA 2013 conference for their hard work in making ISBRA 2013 a successful event. We also thank the students and staff of the UNC Charlotte Department of Bioinformatics and Genomics for providing logistical support and volunteer labor before and during the conference.

We thank the Editors-in-Chief of BMC Bioinformatics and BMC Genomics for providing us with the opportunity to showcase some of the research presented at ISBRA 2013. We thank the National Science Foundation and the North Carolina Biotechnology Center for their sponsorship of the conference, along with corporate sponsors CLC Genomics, Inc. and Accelerated Technology Laboratories. Last but not the least, we would like to thank all the ISBRA 2013 authors. The symposium could not continue to thrive without their high quality contributions.

## Declaration

Funding for ISBRA 2013 was provided by the National Science Foundation through award number 1331534 to Cynthia Gibas, by the North Carolina Biotechnology Center, and by donations from corporate sponsors Accelerated Technology Laboratories and CLC Genomics, Inc. The funders had no role in the design, collection, analysis or interpretation of data, in preparation of this manuscript, or in the decision to submit this manuscript for publication.

This article has been published as part of *BMC Bioinformatics *Volume 15 Supplement 13, 2014: Selected articles from the 9th International Symposium on Bioinformatics Research and Applications (ISBRA'13): Bioinformatics. The full contents of the supplement are available online at http://www.biomedcentral.com/bmcbioinformatics/supplements/15/S13.
